# Retraction Note: Effect of a single intra-articular high molecular weight hyaluronan in a naturally occurring canine osteoarthritis model: a randomized controlled trial

**DOI:** 10.1186/s13018-023-03806-5

**Published:** 2023-04-28

**Authors:** J. C. Alves, Ana Margarida Moniz Pereira dos Santos, Patrícia Jorge, Catarina Falcão Trigoso Vieira Branco Lavrador, L. Miguel Carreira

**Affiliations:** 1 Divisão de Medicina Veterinária, Guarda Nacional Republicana (GNR), Rua Presidente Arriaga, 9, 1200-771 Lisbon, Portugal; 2 MED – Mediterranean Institute for Agriculture, Environment and Development, Instituto de Investigação E Formação Avançada, Universidade de Évora, Pólo da Mitra, P. 94, 7006-554 Évora, Portugal; 3 Faculty of Veterinary Medicine, University of Lisbon (FMV/ULisboa), Lisbon, Portugal; 4 Interdisciplinary Centre for Research in Animal Health (CIISA), University of Lisbon (FMV/ULisboa), Lisbon, Portugal; 5 Anjos of Assis Veterinary Medicine Centre (CMVAA), Barreiro, Portugal

**Retraction Note: Journal of Orthopaedic Surgery and Research (2021) 16:290** **https://doi.org/10.1186/s13018-021-02423-4**

The Editor-in-Chief has retracted this article. After publication, concerns were raised regarding potential reuse of the data reported in the article. Specifically:

The article appears to include a subset of data from [1] without an appropriate citation.

The control group data and results appear to be highly similar to those published in the authors' other articles [2–4].

The Hyaluronan group T0 values in Table 4 appear to match those for the Control group in [2–4].

The authors have been contacted to address these concerns. The corresponding author stated that the data are unique to each article. However, based on the extent of data similarities among these articles, the Editor-in-Chief no longer has confidence in the presented data.

J. C. Alves and C. Lavrador do not agree to this retraction. A. Santos, P. Jorge and L. Miguel Carreira have not responded to any correspondence from the editor or publisher about this retraction.
